# Unspecific Findings of Oropharyngeal and Esophageal Dysmotility During Solid Bolus Swallowing

**DOI:** 10.1002/jgh3.70378

**Published:** 2026-03-09

**Authors:** Per Askelöf, Olle Ekberg, Bodil Ohlsson

**Affiliations:** ^1^ Department of Radiology Diagnostics Skåne University Hospital Malmö Sweden; ^2^ Department of Translational Medicine Malmö, Lund University Lund Sweden; ^3^ Department of Clinical Sciences Malmö, Lund University Lund Sweden; ^4^ Department of Internal Medicine Skåne University Hospital Malmö Sweden

**Keywords:** dysphagia, esophageal dysmotility, oropharyngeal dysmotility, swallowing test

## Abstract

**Background and Aim:**

Dysphagia is a common symptom which can be caused by several functional and organic conditions. The aim of the present retrospective study was to re‐evaluate examinations from patients diagnosed with functional dysphagia/hypersensitive esophagus and to evaluate relationship between bolus arrest and symptoms/comorbidities.

**Methods:**

All elective radiological swallowing examinations performed during a prior 2‐year period were identified (*n* = 335). Among those who had undergone tablet swallowing test and gave consent to participate, 42 patients (13%) had received the diagnosis functional dysphagia/hypersensitive esophagus. Medical records were scrutinized for these patients.

**Results:**

After exclusion of 2 patients with inadequate information from the examination, 40 patients (68% women), 55 ± 16 years, weight 74.4 ± 19.7 kg, remained. When re‐evaluating the examinations, the liquid phase revealed that 2 patients had slow contrast passage, 10 patients had some degree of non‐propulsive and/or tertiary contractions, and 6 patients had pharyngeal disturbances with retention and/or aspiration of contrast. Although passage of the tablet to the stomach, 27 patients exhibited a transient tablet stop, which led to symptoms in 16 cases. There was a strong correlation between tablet arrest and symptoms (*p* < 0.001) but not comorbidity (*p* = 0.596).Few other examinations were performed to further evaluate the etiology of dysphagia, although several of the patients had comorbidity in the form of reflux, and systemic diseases such as diabetes and rheumatological‐ and neurological diseases.

**Conclusions:**

This report highlights the needs of improved health care for the patient group suffering from dysphagia and a closer inter‐disciplinary collaboration.

Dysphagia is a common symptom which can depend on oropharyngeal mechanisms of dysphagia, structural lesions in the esophagus, gastro‐esophageal reflux disease (GERD), eosinophilic esophagitis (EoE), and major motor disorders. When all organic reasons to dysphagia are excluded, the diagnosis of functional dysphagia is set to describe the sense of solid and/or liquid foods sticking, lodging, or passing abnormally through the esophagus [1]. However, small abnormalities may be difficult to discover and interpret and may not fulfill the established criteria of major motility disorders [2]. Furthermore, not all centers have access to advanced modalities for a complete oro‐pharyngeal‐esophageal examination. Sometimes, unspecific findings are called functional, to give some sort of name to the patient and health care staff. Our hypothesis was that several of the patients diagnosed as functional dysphagia exhibited organic abnormalities and/or dysmotility. The primary aim of the present study was to re‐evaluate examinations from patients diagnosed with functional dysphagia/hypersensitive esophagus, to better evaluate the diagnostic yield of solid tablet swallowing. Secondary aims were to evaluate the relationship between transient bolus arrest and symptoms and comorbidities.

A search was performed in the autumn of 2024 at the Department of Radiology, Skåne University Hospital, Malmö, to identify all elective radiological swallowing examinations during a prior 2‐year period. Information letter was sent retrospectively to examined patients. Those who did not want to participate had to return a signed form that they declined participation, a so‐called opt‐out procedure. Patients who did not return their forms were included in the study and their medical records were scrutinized.

In total, 335 patients, 192 women (57%), mean age 63 ± 18 (range 18–96) years, underwent a radiological swallowing evaluation. Patients with a clinical history highly suspicious for aspiration were first given iodine contrast (Omnipaque 240 mg I/mL; GE Healthcare, Danderyd, Sweden). If aspiration occurred, the patient did not undergo the following protocol. Patients without a clinical history of aspiration and/or who did not aspirate iodine contrast were given barium sulfate (E‐Z‐HD; EZ EM Canada Inc., Montreal, QC, Canada). This included examination in an erect position in AP/coronal, lateral, and oblique views of pharynx/esophagus. The patients were then examined recumbent with a less opaque barium solution (Polibar Plus; EZ EM Canada Inc.; 40% weight/volume) focused on esophageal peristalsis. Since solid bolus arrest is usually due to esophageal narrowing or dysmotility, patients were carefully interviewed for any symptoms of obstruction. Those who reported some degree of obstruction were qualified for an additional examination with solid bolus swallowing. The patients were given a solid bolus in the form of a Novalucol tablet (Cooper Consumer Health B, Paris, France), diameter 14 mm and height 6 mm, according to Ekberg and van Westen [3]. The tablet was swallowed with Polibar. Novalucol is a common antacid in Sweden with the active substance calcium carbonate and magnesium hydroxide, which appears radiolucent at fluoroscopy.

Pharyngeal and esophageal abnormalities such as paresis, diverticula, webs, and strictures/tumors were registered as well as retention and aspiration of liquid contrast. Slow contrast passage, non‐propulsive, or tertiary contractions were registered. Focus on solid bolus/tablet examinations were classified into either: (1) no tablet arrest (2) tablet arrest or (3) tablet passage not mentioned or impossible to determine. In case of arrest, localization was registered. Presence of symptoms like experience at home was categorized depending on relationship with tablet arrest. Comorbidity and other examinations performed were registered from medical records. Fisher's exact test was used to calculate correlations between tablet arrest and symptoms/comorbidity.

Out of the patients who were intended to undergo tablet swallowing and gave consent to participate, 42 patients (13%) had received the diagnosis functional dysphagia/hypersensitive esophagus, depending on inconclusive findings without possibility to define a certain diagnosis. Small motility disturbances were found not fulfilling the criteria for any known dysmotility disease, and/or the tablet passed to the stomach after a small arrest. Most of the cases experienced well‐known symptoms during the examination.

After scrutinizing the medical records, 2 patients were excluded due to inadequate information from the radiological examination. Thus, 40 patients, 27 women (68%), mean age 55 ± 16 years, range 18–88 years, weight 74.4 ± 19.7 kg, body mass index (BMI) 26.3 ± 6.5 kg/m^2^, remained to be included for further evaluation. Among these, several suffered from different comorbidities associated with dysmotility, the most common being GERD (*n* = 10), hypothyroidism (*n* = 5), diabetes (*n* = 4), rheumatological diseases (*n* = 4), esophagitis/Barrett's disease (*n* = 4), neurological diseases (*n* = 2), neuropsychiatric diagnosis (*n* = 2), asthma bronchialis (*n* = 2), ulcer (*n* = 2), lung/tongue malignancy (*n* = 2), and obesity (*n* = 2). Only 1 patient was diagnosed with irritable bowel syndrome (IBS) or any other functional disorder. The most common medical treatments were proton pump inhibitors (PPI) (*n* = 12), nonsteroidal anti‐inflammatory drugs (NSAID) (*n* = 8), calcium channel blockers (*n* = 5), beta blockers (*n* = 4), paracetamol (*n* = 4), statins (*n* = 4), levothyroxine (*n* = 4), and angiotensin‐converting enzyme (ACE) inhibitors (*n* = 3). Further drugs were used sporadically among the participants.

When re‐evaluating the 40 examinations, the liquid phase revealed that 2 patients had slow passage of contrast, 10 patients had some degree of non‐propulsive and/or tertiary contractions, and 6 patients had pharyngeal swallowing disturbances with retention and/or aspiration of contrast. The solid bolus examination was not performed in 2 cases since 1 patient refused and the other ingested food instead. Furthermore, passage information was missing in 5 patients. Although the tablet passed to the stomach, as many as 27 patients (82% of 33 examinations) exhibited a transient stop of the tablet (Table 1), which led to symptoms in the patients in 16 cases (59%). The connection between transient arrest and symptoms was not mentioned in 14 cases, and symptoms were present in 6 cases without described arrest. There was a strong correlation between tablet arrest and symptoms (*p* < 0.001), but not with comorbidity (*p* = 0.596). There was consensus among the authors regarding the findings.

**TABLE 1:** Findings in the 40 patients examined by solid swallowing test.

	Pathological findings/bolus arrest	Normal passage	Missing information	Not performed
Findings				
Tablet passage	27^a^	6	5	2
Pharyngeal retention/aspiration	6	34		
Contrast passage	2	38		
Motility	10	30		

	Pathological findings	Normal findings	Missing information	
Complementary examinations				
Gastroscopy	6	22	12	
Histopathological examination	4	11		
Esophageal manometry	1	4		
pH measurement	1	3		
Esophageal X‐ray	1	1		

*Note:* Findings are the summary of the information obtained after scrutinizing the medical records.

^a^ 26 esophageal arrest (2 proximal esophagus, 14 middle of esophagus, and 7 distal esophagus, and 3 without description of localization), and 1 without description of localization for the arrest.

When searching for other examinations performed, only 5 of 40 included patients (12%) had been examined by esophageal manometry, 4 by pH measurement, and 2 with esophageal X‐ray, where pathological findings were described in 1 of each examination. Gastroscopy was performed in 28 patients (70%), with pathological findings of ulcer, esophagitis, or Barrett's esophagitis in 6 cases. Histopathological examination was performed in biopsies from 15 cases, with description of 4 cases of inflammation but no EoE (Table 1).

The main outcome of the above‐mentioned findings is that small/minor changes in swallowing and motility may be difficult to interpret in clinical praxis. Several systemic diseases are associated with gastrointestinal dysmotility. Although major dysmotility was excluded, the comorbidity of systemic diseases affecting motility, such as diabetes, multiple sclerosis, Parkinson's disease, and rheumatological diseases suggests any form of neurological and/or muscular dysfunction. The symptoms and minor changes may be early signs of dysmotility, not yet fulfilling the established criteria for major dysmotility disorders [2]. Hypercontractile esophagus is a heterogenous motility disorder, often associated with dysphagia. Provocative testing during high‐resolution manometry (HRM) and FLIP panometry enhance the ability to detect hypercontractile esophagus, reproduce symptoms, and understand the pathophysiology behind the patients' symptom [4].

The main components of swallowing physiology contain three functional domains, namely, oral, pharyngeal, and esophageal domains. The esophageal domain of the swallowing examination focuses on the degree of esophageal bolus clearance and is not a complete examination of esophageal motility [5, 6]. Nevertheless, there is an association between oropharyngeal and esophageal motility [7]. The most informative examination to be used must depend on the symptoms and needs for the patients and should be preceded by a careful discussion between the clinician, radiologist, and speech and language therapist [5]. On the contrary, the solid bolus of a Novalucol tablet may be too big, leading to arrest and symptoms in healthy people. The correlation between tablet arrest and symptoms like experience at home suggests passage obstacles at meal intake.

Disturbances of brain‐gut interaction (DBGI) are common in the population. Often, patients suffer from more than one of these disorders; IBS being the most common with a prevalence of 4.1% worldwide [8]. To diagnose functional dysphagia in patients with a sense of abnormal passage of solid and/or liquid food, organic disorders must be excluded, including esophageal mucosal or structural abnormality, GERD, EoE, and major motor disorders. Functional dysphagia is estimated to be the least common functional esophageal disorder [1], and the overall worldwide prevalence is 3.2% [8]. Thus, the prevalence of 13% among symptomatic patients undergoing swallowing examination seems unexpectedly high. When the comorbidity of other organic and systemic diseases is present, patients should not be diagnosed with any functional disorder until any organic dysmotility has been completely excluded [9]. Discrete events during the oropharyngeal swallow may reveal crucial abnormalities that are easy to overlook if not properly evaluated according to standardized procedures with a validated protocol using thin and thick liquids, such as the modified barium swallow study (MBSS) [5]. Several of the current patients with dysphagia had objective signs of reflux, which are known to be associated with esophageal dysmotility. This comorbidity further emphasizes the importance of correct examination and diagnosis. The concomitant presence of GERD was, in most cases, not obvious for the examiner. Neither was the association between GERD and dysmotility considered by the referred clinician. Furthermore, EoE was only ruled out in 38% of the patients.

It is of great difference for the patient to be informed that the dysmotility is in accordance with the original disease, like diabetes with other known complications, or related to their reflux symptoms, than to be informed about another poorly defined functional diagnosis. Further, the treatment of esophageal dysmotility may lead to modified dietary food habits, which is not the treatment of choice for patients with functional disorders [1]. Thus, correct diagnosis is crucial for the patients' care and a misdiagnosis can have great negative effects on the patients' health and quality of life.

There are several limitations of this retrospective study. The solid bolus in the form of a tablet may not represent a solid meal, depending on differences in weight, shape, and texture. The advantage of using a tablet is its simplicity and reproducibility, and the lower risk of harmful aspiration or retention in patients with severe dysmotility or obstructions.

In conclusion, the complex etiology, symptomatology, and examination procedures concerning dysphagia demand a close inter‐disciplinary collaboration. This brief report highlights the need for improved health care for this patient group suffering from dysphagia.

## Funding

The study was funded by Development Foundation of Region Skåne, project 2025‐2026‐2024‐2293.

## Ethics Statement

Swedish Ethical Review Authority, no. 2024‐03651‐01, approval date 03/07/2024.

## Conflicts of Interest

The authors declare no conflicts of interest.

## Data Availability

The data that support the findings of this study are available on request from the corresponding author. The data are not publicly available due to privacy or ethical restrictions.
